# Effects of Chitosan and Duck Fat-Based Emulsion Coatings on the Quality Characteristics of Chicken Meat during Storage

**DOI:** 10.3390/foods11020245

**Published:** 2022-01-17

**Authors:** Dong-Min Shin, Yea-Ji Kim, Jong-Hyeok Yune, Do-Hyun Kim, Hyuk-Cheol Kwon, Hyejin Sohn, Seo-Gu Han, Jong-Hyeon Han, Su-Jin Lim, Sung-Gu Han

**Affiliations:** Department of Food Science and Biotechnology of Animal Resources, Konkuk University, Seoul 05029, Korea; jeff.shin90@gmail.com (D.-M.S.); dpwl961113@konkuk.ac.kr (Y.-J.K.); skyun0423@konkuk.ac.kr (J.-H.Y.); secret311@konkuk.ac.kr (D.-H.K.); rnjs1024@konkuk.ac.kr (H.-C.K.); sonhjin123@konkuk.ac.kr (H.S.); tjrn8854@konkuk.ac.kr (S.-G.H.); hyeon4970@konkuk.ac.kr (J.-H.H.); tnwlsdl1110@konkuk.ac.kr (S.-J.L.)

**Keywords:** duck fat, chitosan, edible coating, chicken meat, shelf-life

## Abstract

Chicken meat is a popular food commodity that is widely consumed worldwide. However, the shelf-life or quality maintenance of chicken meat is a major concern for industries because of spoilage by microbial growth. The aim of this study was to evaluate the effects of chitosan and duck fat-based emulsion coatings on the quality characteristics and microbial stability of chicken meat during refrigerated storage. The coated chicken meat samples were as follows: control (non-coated), DFC0 (coated with duck fat), DFC0.5 (coated with duck fat and 0.5% chitosan), DFC1 (coated with duck fat and 1% chitosan), DFC2 (coated with duck fat and 2% chitosan), and SOC2 (coated with soybean oil and 2% chitosan). The results showed that the apparent viscosity and coating rate were higher in DFC2 than in other groups. Physicochemical parameters (pH, color, and Warner–Bratzler shear force) were better in DFC2 than those in other groups during 15 days of storage. Moreover, DFC2 delayed lipid oxidation, protein deterioration, and growth of microorganisms during storage. These data suggest that chitosan-supplemented duck fat-based emulsion coating could be used to maintain the quality of raw chicken meat during refrigerated storage.

## 1. Introduction

The consumption of chicken meat has increased over recent decades because of its low-cost, low-fat content, high nutritional value, and unique flavor [1]. However, chicken meat is a perishable product because it enables the growth of spoilage and pathogenic microorganisms [2]. This is because of its high moisture and protein contents and high pH value. The shelf-life of chicken meat is as short as 3–5 days in a refrigerator [3]. Hence, the chicken meat industry is interested in extending the shelf-life of raw chicken meat.

In recent years, edible coating technology has received attention for improving food quality and shelf-life. Edible emulsion coatings are described as a thin and continuous layer of edible biomaterial that may be formed or placed on or between foods [4]. These coating biomaterials are mainly derived from natural materials, including proteins (e.g., gelatin, whey, and zein), polysaccharides (e.g., chitosan and alginate), and lipids (e.g., soybean oil and sunflower oil) [5,6]. However, protein and polysaccharide-based coating materials are highly vulnerable to moisture and are not suitable for water-resistant coatings [7]. Among various coating materials, lipid-based edible coatings provide a better moisture barrier and protection for foods, and vegetable oils and waxes are the main components of edible coatings [6]. However, the use of vegetable oils causes lipid oxidation due to the high levels of unsaturated fatty acids, as the predominant fatty acid in vegetable oils is linoleic acid [8]. According to a previous study, sunflower oil–chitosan edible films for pork hamburgers were more vulnerable to oxidation than non-coated samples [9]. Moreover, for emulsion coatings, rheological properties, such as apparent viscosity and yield stress, are important for determining the coating quality [7]. Linoleic acid-rich lipid products have poorer rheological and textural properties than oleic acid-rich lipid products [10].

Duck meat is well-known for its unique flavor and aroma, and high nutritional values such as essential amino acids and unsaturated fatty acids [11]. Duck fat is usually obtained as a by-product during duck meat production [12]. Duck fat contains high levels of unsaturated fatty acids (64.51%), including oleic acid (48.7%) and linoleic acid (15.8%), as well as low levels of saturated fatty acids (28.53%) compared with other animal fats (e.g., beef fat and pork fat) [13]. Hence, duck fat intake has the potential to provide health benefits to humans by decreasing the risk of cardiovascular diseases [14,15]. In addition, unlike linoleic acid-rich oil, the presence of oleic acid in duck fat can delay lipid oxidation due to its resistance to oxidation [16]. In addition, the high oleic acid content of duck fat can provide strong physical and thermal resistance in lipid-based products [10]. Like duck fat, olive oil is also rich in oleic acid. However, olive oil is not a suitable material for manufacturing an edible coating solution due to its dark color and price, compared to other oils [17,18]. Considering the cost efficiency, duck fat is a cheaper source for an edible coating solution than olive oil [13].

Edible coatings are a new approach to controlling microbial growth, and thereby improve the shelf-life and safety of meat, fish, and poultry products [19]. In fact, lipid-based edible coatings are insufficient to control microorganisms, and, thus, the use of antimicrobial agents is required [9]. Chitosan is made by the deacetylation of chitin and is a versatile biopolymer. Chitosan is used as a natural preservative for edible coating manufacturing because of its strong antimicrobial properties against several foodborne microorganisms [3]. Many studies have reported that chitosan-added edible coatings can extend the shelf-life of fresh fruits and vegetables [20,21]. Additionally, chitosan-added edible coatings can be applied to fresh poultry, meat, and fish products. For example, the effects of chitosan coatings and gamma irradiation on chicken meat [22], chitosan–gelatin edible coatings with nisin and grape seed extract on fresh pork [23], chitosan coatings incorporated with lactoperoxidase on trout [24], and chitosan–soybean oil emulsion coatings on eggs [25] have been reported.

There are limited publications that provide practical and effective coating techniques for the chicken meat industry. Therefore, the objective of this study was to evaluate the effects of chitosan and duck fat-based edible coatings on the quality characteristics and microbial stability of chicken meat during refrigerated storage (at 4 °C for 15 days).

## 2. Materials and Methods

### 2.1. Materials

Chicken meat and soybean oil (SO; Beksul, Incheon, Korea) were purchased from a local market. Duck fat (DF) was kindly provided by Taekyung Nongsan (Seoul, Korea). Chitosan (molecular weight of 310–375 kDa, acid-soluble, and coarse ground flakes and powder from crustacean shells), lecithin, Tween^®^ 80 (polyoxyethylene-20 sorbitan monooleate), thiobarbituric acid (TBA), chloroform, bromocresol green, methyl red, boric acid, sulfuric acid, and acetic acid were obtained from Sigma-Aldrich Co. (St. Louis, MO, USA).

### 2.2. Preparation of Coating Solution and Coating of Chicken Meat

The edible coating solution was prepared as previously described [25]. Briefly, chitosan (final pH of 4.52) was prepared by dissolving chitosan in 1% acetic acid (*v*/*v*) solution (i.e., 0.5, 1.0, and 2.0 g of chitosan/100 mL acetic acid (*w*/*v*)). The chitosan solution and DF were mixed at a ratio of 40:60 by adding the Tween^®^ 80 emulsifier. This chitosan/DF mixture was blended for 3 min at low speed, followed by blending for 6 min at high-speed using a hand blender (Tefal Co., Ltd., Mayenne, France). The mixture was homogenized at 20,000 rpm for 3 min using a homogenizer (DAIHAN Scientific Co., Ltd., Gangwon, Korea).

To coat the chicken meat, the meat samples were immersed in the coating solution for 2 min under magnetic stirring at 800 rpm. Samples were then placed in a biological hood for 2 h at 25 ± 2 °C to form an edible coating. The coated sample was sealed in a polyethylene bag and stored at 4 ± 1 °C. Sample analyses were performed on days 0, 3, 5, 7, 10, and 15 of refrigerated storage. The total number of chicken breast meat slices used for physicochemical and microbiological analyses was 288. The control and treatment groups were prepared as follows: control (NC, non-coated), DFC0 (coated with duck fat), DFC0.5 (coated with duck fat and 0.5% chitosan), DFC1 (coated with duck fat and 1% chitosan), DFC2 (coated with duck fat and 2% chitosan), and SOC2 (coated with soybean oil and 2% chitosan).

### 2.3. Apparent Viscosity of Coating Solution

The apparent viscosity of the coating solution was measured using a rheometer (model MCR 92, Anton Paar, Graz, Austria) at 25 °C, and the data were collected between shear rates of 0.1 and 100 Hz (*n* = 3/group). The results are expressed in units of Pascal-seconds (Pa-s). The data were analyzed using an Anton Paar RheoCompass Ver. 1.25.

### 2.4. Coating Rate of Samples

The coating rate was determined as described previously [26]. The coating rate (%) of samples was calculated as (weight of coated chicken meat (g)—weight of raw chicken meat (g))/weight of coated chicken meat (g) × 100.

### 2.5. pH and Color Measurements of Chicken Meat

The pH of the coated chicken meat was determined using a pH meter (LAQUA, Horiba, Ltd., Kyoto, Japan). Briefly, 5 g of sample and 20 mL distilled water were homogenized at 10,000 rpm for 30 s using a homogenizer (DAIHAN Scientific Co., Ltd., Gangwon, Korea), and the pH of the homogenate was measured. Color was measured on the surface of the coated samples using a CR-210 colorimeter (Minolta Camera Co., Ltd., Osaka, Japan) with standard white calibration plates. The data were expressed as L* (lightness), a* (redness), and b* (yellowness) values.

### 2.6. Warner–Bratzler Shear Force (WBSF)

The WBSF of the chicken meat was measured using a TA-XT2i texture analyzer (Stable Micro Systems Ltd., Godalming, UK) equipped with a Warner–Bratzler shear attachment (V-type blade set). Samples were cut to sizes of 2.0 × 2.0 cm (*n* = 8). The WBSF was analyzed under the following conditions: a test speed of 2.0 mm/s, a post-test speed of 4.0 mm/s, and a distance of 25.0 mm. The maximum force required to shear through the samples was determined and analyzed as WBSF.

### 2.7. Lipid Oxidation

The lipid oxidation of coated chicken meat was evaluated by measuring the development of thiobarbituric acid reactive substances (TBARS) according to a previously described method [27]. Briefly, the coated sample (10 g) was blended with distilled water (50 mL), and then the mixture was homogenized for 2 min using a Model AM-7 homogenizer (Nissei Co., Ltd., Tokyo, Japan). The homogenate was transferred to a distillation flask and 47.5 mL of distilled water, 2.5 mL of 4 N HCl solution, and 1 mL of antifoam agent (KMK-73, Shin-Etsu Silicone Co., Ltd., Seoul, Korea) were added to it. The mixture was distilled, and 40 mL of the distillate was collected. Then, 5 mL of the collected sample and 5 mL of TBA reagent (0.02 M in 90% acetic acid) were mixed in a test tube and heated at 95 °C for 30 min. After cooling, the absorbance of the samples was measured at 538 nm for TBARS measurements using a UV/VIS spectrophotometer (Optizen 2120 UV Plus, Mecasys Co., Ltd., Daejeon, Korea).

### 2.8. Volatile Basic Nitrogen (VBN)

The volatile basic nitrogen (VBN, mg%) content was determined using the Conway microdiffusion method, as reported previously [28]. In brief, 5 g of the coated chicken meat sample was mixed with 20 mL of distilled water. The mixtures were homogenized at 10,000 rpm for 1 min using a homogenizer (Model AM-7, Nihonseiki Kaisha Ltd., Tokyo, Japan) and filtered using Whatman No. 1 filter paper (Whatman International, Maidstone, UK). After filtering, 30 mL of distilled water was added. Then, 1 mL of the filtered sample and 1 mL of 50% K_2_CO_3_ solution were added to the outer section, and 100 μL of indicator (1:1 = 0.066% bromocresol green in ethanol–0.066% methyl red in ethanol) and 1 mL of 0.01 N H_3_BO_3_ were added to the inner section of the Conway microdiffusion cells. The cells were incubated for 90 min at 37 °C, and the solution in the inner section was titrated with 0.02 N H_2_SO_4_ solution.

### 2.9. Microbiological Analysis

Microbiological evaluation was performed on days 0, 3, 5, 7, 10, and 15 of storage. Briefly, 25 g of coated chicken meat sample was mixed using a stomacher (Masticator Paddle Blender, IUL Instrument, Barcelona, Spain) with 225 mL of 0.1% peptone water for 2 min. The mixtures were serially diluted with 0.1% peptone water. The total viable count (TVC) and *Listeria* spp. were counted on plate count agar (Merck, Darmstadt, Germany) and Oxford agar (Oxoid Ltd., Hampshire, UK), and each agar was incubated at 37 °C for 24 h. *Escherichia coli*, coliforms, molds, and yeasts were counted using Petrifilm (3M, St. Paul, MN, USA). *E. coli* and coliforms were incubated at 36 °C for 24 h, and molds and yeasts were incubated at 25 °C for 5 days. The results are expressed as log CFU/g.

### 2.10. Statistical Analysis

Each experiment was performed in triplicate and the data are expressed as mean ± standard deviation (SD). A two-way analysis of variance (ANOVA) followed by Duncan’s multiple range test (*p* < 0.05) was conducted using SPSS Ver. 24.0 (SPSS Inc., Chicago, IL, USA) for assessing significant differences.

## 3. Results and Discussion

### 3.1. Apparent Viscosity of Coating Solution and Coating Rate

**Figure 1:**
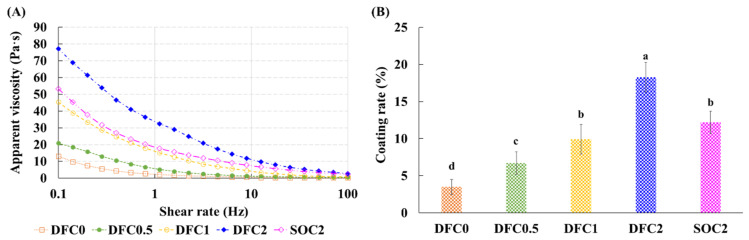
Apparent viscosity and coating rate of the coating solution. (**A**) Apparent viscosity (*n* = 3) and (**B**) coating rate (*n* = 3). DFC0, coated with duck fat, with no chitosan; DFC0.5, coated with duck fat and 0.5% chitosan; DFC1, coated with duck fat and 1% chitosan; DFC2, coated with duck fat and 2% chitosan; SOC2, coated with soybean oil and 2% chitosan. Bars with the different letter are significantly different (*p* < 0.05), and the error bars indicate SD.

The apparent viscosities of the coating solutions are shown in Figure 1A. The apparent viscosity of the coating solution was significantly affected by the type of lipid and the addition of chitosan, where DFC2 exhibited the highest apparent viscosity among the groups (*p* < 0.05). This can be explained by the melting point of duck fat. In a previous study, duck fat-added margarine had higher apparent viscosity than soybean oil-added margarine due to the higher melting point of duck fat (6.21 °C) than that of soybean oil (−22.59 °C) [10]. The chitosan content also affected the apparent viscosity of the coating solution. This can be explained by the degree of chain entanglement in the coating solution. As the polymer concentration increases, the freedom of movement of polymer chains is restricted because of the correspondingly greater entanglement [29].

The coating rate of the coating solution showed trends similar to those of apparent viscosity (Figure 1B). The coating rate increased as the amount of chitosan increased in the duck fat. Duck fat had a higher coating rate than soybean oil. More specifically, the coating rate of DFC2 was higher than that of other coating solution groups (*p* < 0.05). This may be due to an increase in the apparent viscosity of the coating solution. A high viscosity of the solution can lead to a more stable shape, which leads to a higher coating yield [30]. In addition, polysaccharides, such as chitosan and dietary fiber have a high water holding capacity, which can enhance the emulsifying capacity, thereby increasing viscosity [31,32].

### 3.2. pH and Color of Coated Chicken Meat

Chicken meat is more prone to rapid bacterial deterioration than pork and beef because raw chicken meat generally has a higher pH (0.2–0.4 higher than raw pork and beef) [33]. In addition, the changes in the pH values of chicken meat are highly related to microbial balance, which can lead to a low shelf-life [34]. The pH values of coated chicken meat during refrigerated storage are presented in Table 1. As expected, the pH of all samples tended to increase with storage until day 10 (*p* < 0.05). This increase was significantly higher in the NC group than in other groups (*p* < 0.05). This may be due to the antimicrobial activity of chitosan in the coating solution. A previous study reported that the antibacterial properties of chitosan in coated samples were associated with the lower pH values of samples [35]. Moreover, when chicken meat becomes spoilt, VBN values tend to increase due to the production of NH_3_ along with other volatile amines [36]. Therefore, the higher pH of NC could be explained by faster spoilage than that of the coated groups.

Color is considered by consumers as the most important factor in the marketability of meat and poultry. Table 1 shows the L* (lightness), a* (redness), and b* (yellowness) values of the coated chicken meat during storage. Both the storage period and the presence of coating affected the color of the samples (*p* < 0.05). The L* values of all samples decreased during storage (*p* < 0.05). The coated samples showed higher L* values than the NC group (*p* < 0.05). Using chitosan-based emulsions for the coating solution could lead to an increase in the L* value of the samples. When the chitosan emulsion forms, the turbidity and opacity of the solution can increase, resulting in increased lightness [37]. Similar results were reported where a chitosan–essential oil solution increased the L* value of coated chicken meat [34]. The changes in a* and b* values are highly associated with the formation of metmyoglobin, which forms by the oxygenation of myoglobin [38]. As the storage period increased, the a* values of all samples decreased, and b* values increased. (*p* < 0.05). DFC1, DFC2, and SOC2 showed higher a* values and lower b* values than the NC group during the storage period (*p* < 0.05). This was probably due to the inhibition of myoglobin oxidation by the antioxidant activity of chitosan. Cooked pork chops coated with chitosan and bamboo vinegar effectively maintain their initial a* and b* values during storage because chitosan has high antioxidant properties and can maintain meat color because of its ability to act as a chelator of transition metal ions [39]. Overall, the chitosan and duck fat-based emulsion coating may be a good option for inhibiting the discoloration of chicken meat during refrigerated storage.

### 3.3. Warner–Bratzler Shear Force of Coated Chicken Meat

**Figure 2:**
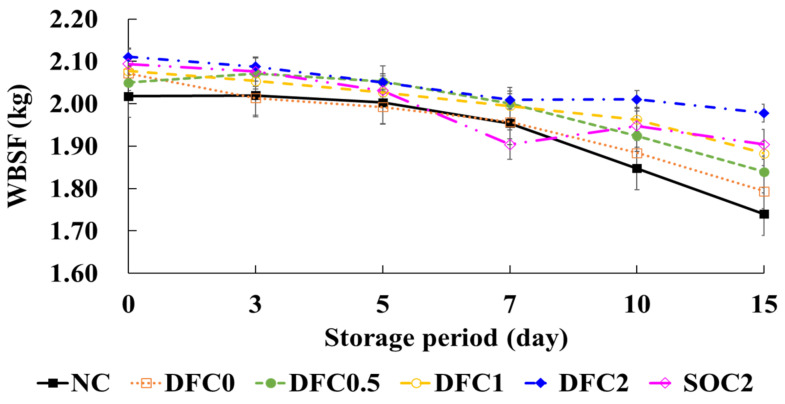
Warner–Bratzler shear force (WBSF) of coated chicken meat during storage at 4 ± 1 °C for 15 days. NC, non-coated; DFC0, coated with duck fat, with no chitosan; DFC0.5, coated with duck fat and 0.5% chitosan; DFC1, coated with duck fat and 1% chitosan; DFC2, coated with duck fat and 2% chitosan; SOC2, coated with soybean oil and 2% chitosan. The error bars indicate SD (*n* = 3).

The WBSF values are related to the tenderness of meat samples, which is a critical organoleptic property that affects consumer preference [40]. In our study, the WBSF values of coated chicken meat were significantly affected by the presence of edible coatings (Figure 2). WBSF values gradually decreased during storage (*p* < 0.05). DFC2 exhibited the highest WBSF values during storage, whereas WBSF values were lower in the NC group than those in other groups (*p* < 0.05). This result might be attributable to the deterioration of proteins by microorganisms. The WBSF values of samples can decrease due to the degradation of proteins in meat, mainly caused by bacterial or enzymatic processes as storage progresses [41]. Thus, a higher microorganism count in NC may affect the decrease in the WBSF values of meat samples during storage. Collectively, our data suggest that the chitosan–duck fat edible coating for chicken meat can contribute to maintaining meat tenderness by inhibiting microorganisms.

### 3.4. TBARS Values and VBN of Coated Chicken Meat

**Figure 3:**
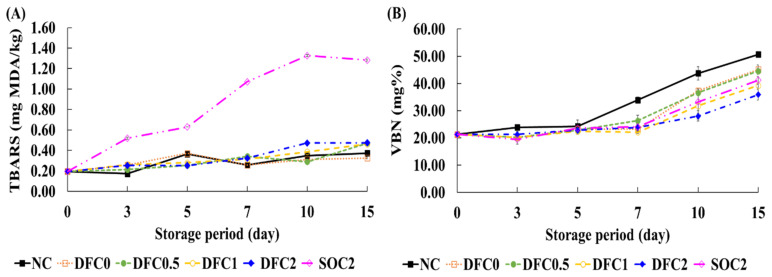
TBARS and VBN values of coated chicken meat during storage at 4 ± 1 °C for up to 15 days. (**A**) Thiobarbituric acid reactive substances (TBARS) and (**B**) volatile basic nitrogen (VBN) values were determined during storage at 4 ± 1 °C for up to 15 days. NC, non-coated; DFC0, coated with duck fat, with no chitosan; DFC0.5, coated with duck fat and 0.5% chitosan; DFC1, coated with duck fat and 1% chitosan; DFC2, coated with duck fat and 2% chitosan; SOC2, coated with soybean oil and 2% chitosan. The error bars indicate SD (*n* = 3).

Shelf-life and the quality of meat are highly associated with lipid oxidation and protein deterioration during storage [42]. TBARS values are used for measuring the formation of secondary oxidation products, including malondialdehyde, alkenals, and alkadienals [43]. Variations in the TBARS values of the meat samples are shown in Figure 3A. The difference in TBARS values between DFC treatments and SOC2 can be explained by the fatty acid profiles of duck fat and soybean oil in the coating solution. Duck fat is more stable against lipid oxidation than soybean oil during storage because the main fatty acids in duck fat and soybean oil are oleic acid and linoleic acid, respectively [10]. The oxidative stability of oleic acid in edible oils is almost 10-times greater than that of linoleic acid [16]. Therefore, the higher resistance to oxidation of duck fat could be more suitable as an edible coating material than soybean oil.

The VBN value is an important indicator of protein deterioration in meat and meat products [44]. VBN mainly includes ammonia and primary, secondary, and tertiary amines. In general, the VBN value is an indicator of meat spoilage, particularly when it exceeds 25 mg% [34]. The VBN data of the chicken meat samples during storage are presented in Figure 3B. VBN values for all samples increased during storage (*p* < 0.05), while DFC2 had significantly lower VBN values among all groups (*p* < 0.05). The VBN values of most samples were over the standard point (25 mg%) on day 10, while DFC2 showed VBN values over the standard point (25 mg%) on day 15. The increase in VBN is related to the hydrolysis of proteins to amino acids, peptides, biogenic amines, inorganic nitrogen, and the increasing contents of volatile bases due to enzymes and microorganisms during storage [45]. Thus, lower microbial growth might be expected in the DFC groups, particularly in the DFC2 group.

### 3.5. Growth of Microorganisms on Coated Chicken Meat

The results of TVC, *E. coli*, coliforms, *Listeria* spp., molds, and yeasts are shown in Table 2. Meat decay is generally defined when the TVC exceeds 7 log CFU/g [46]. The TVC of DFC2 was significantly lower than that of other groups during storage (*p* < 0.05), and only DFC2 did not exceed the standard point for meat spoilage until the end of the storage period. The abundance of *E. coli* and coliforms are important hygienic quality indicators for meat and meat products [47]. The microbial counts increased during storage in all groups, and the rate of this increase was significantly lower in DFC2 than that in NC after 15 days of storage. The meat and meat product surfaces are considerably susceptible to mold and yeast growth, which are related to spoilage and have negative effects on organoleptic properties and safety [34]. At the beginning of storage, mold and yeast were not detected in any sample. After 3 days, mold and yeast were detected in all samples, with NC showing the highest counts of mold and yeast at the end of storage (*p* < 0.05). DFC2 showed significantly higher growth inhibitory effects against mold and yeast during storage (*p* < 0.05). *Listeria* spp. are foodborne pathogens in meat and meat products and have increasingly proliferated despite improvements in control measures [48]. *Listeria* spp. were not detected in any samples until day 3, whereas this pathogen was detected on day 5 only in DFC0 and DFC0.5. Meanwhile, *Listeria* spp. were not detected in DFC2 and SOC2 until the end of storage. These data indicate that chitosan inhibits *Listeria* spp. Overall, DFC2 showed the highest antimicrobial activities against TVC, *E. coli*, coliforms, *Listeria* spp., molds, and yeasts during storage. This was due to the strong antimicrobial properties of chitosan. Previous studies have shown that edible coatings with chitosan can inhibit microorganisms in meat and meat products [22,49]. Overall, the 2% chitosan-added duck fat edible coating can improve the shelf-life of chicken meat by inhibiting the growth of microorganisms during refrigerated storage.

## 4. Conclusions

In this study, the effects of chitosan and duck fat-based emulsion coating on the quality characteristics and microbial stability of chicken breast meat were investigated. The duck fat-based coating solution showed higher apparent viscosity than that of the soybean oil-based coating solution, which resulted in a high coating rate for chicken meat. The physicochemical properties, including pH, color, and WBSF value of the DFC2 group (chicken meat coated with duck fat and 2% chitosan) improved significantly compared to those of other groups (*p* < 0.05). The DFC2 group showed lower lipid oxidation (TBARS value) and protein deterioration (VBN value) during refrigerated storage over 15 days. Furthermore, DFC2 was effective at inhibiting the growth of microorganisms, including TVC, *E. coli*, coliforms, *Listeria* spp., molds, and yeasts during storage. Lower lipid oxidation and protein deterioration in DFC2 were owing to the higher apparent viscosity and coating rate in duck fat compared to soybean oil. Here, the higher viscosity and coating rate in DFC2 were probably due to the higher melting point of duck fat. In addition, the higher coating rate of DFC2 made more chitosan concentrations on the coated samples and that resulted in the extending shelf-life of chicken meat. Our data suggest that chitosan/duck fat-based edible coatings can be used to maintain the quality of raw chicken meat during refrigeration. This edible coating solution could be further studied regarding the sensory properties of coated products and its application in a variety of foods, such as meat products, vegetables, and fruits.

## Figures and Tables

**Table 1:** pH and color of coated chicken meat during storage at 4 ± 1 °C for up to 15 days.

Parameter	Treatment ^1)^	Storage Period (Days)					
		0	3	5	7	10	15
pH	NC	5.99 ± 0.01 ^Ad^	5.92 ± 0.01 ^Ae^	5.98 ± 0.02 ^Ad^	6.20 ± 0.01 ^Aa^	6.14 ± 0.01 ^Ab^	6.03 ± 0.01 ^Ac^
	DFC0	5.86 ± 0.01 ^Cd^	5.86 ± 0.03 ^Bd^	5.92 ± 0.01 ^Bc^	5.85 ± 0.01 ^Cd^	6.08 ± 0.01 ^Ba^	6.00 ± 0.01 ^Bb^
	DFC0.5	5.88 ± 0.01 ^Bb^	5.90 ± 0.01 ^Ab^	5.84 ± 0.01 ^Dc^	5.92 ± 0.01 ^Ba^	5.94 ± 0.01 ^Da^	5.89 ± 0.02 ^Cb^
	DFC1	5.81 ± 0.01 ^Ee^	5.85 ± 0.01 ^Bc^	5.87 ± 0.03 ^Cb^	5.86 ± 0.01 ^Cbc^	5.90 ± 0.01 ^Ea^	5.81 ± 0.01 ^Dd^
	DFC2	5.87 ± 0.01 ^Ccd^	5.90 ± 0.01 ^Abc^	5.94 ± 0.01 ^Bab^	5.83 ± 0.06 ^Cd^	5.97 ± 0.03 ^Ca^	5.79 ± 0.01 ^Ee^
	SOC2	5.84 ± 0.01 ^De^	5.87 ± 0.01 ^Bcd^	5.97 ± 0.01 ^Ab^	5.85 ± 0.02 ^Cde^	5.98 ± 0.01 ^Ca^	5.88 ± 0.01 ^Cc^
L*	NC	58.68 ± 2.90 ^Ba^	57.51 ± 5.34 ^Bab^	57.46 ± 2.38 ^Bab^	56.26 ± 2.91 ^Bab^	56.17 ± 4.85 ^Bab^	54.79 ± 2.61 ^Bb^
	DFC0	62.00 ± 3.37 ^Aa^	61.17 ± 2.48 ^Aa^	60.32 ± 3.75 ^Aab^	58.39 ± 2.84 ^ABbc^	58.33 ± 2.15 ^ABbc^	57.34 ± 3.09 ^Ac^
	DFC0.5	61.84 ± 2.25 ^Aa^	59.91 ± 4.53 ^ABab^	59.87 ± 3.28 ^Aab^	59.42 ± 3.41 ^Aab^	58.46 ± 3.48 ^ABb^	58.06 ± 2.51 ^Ab^
	DFC1	62.04 ± 5.08 ^Aa^	61.81 ± 3.38 ^Aa^	60.73 ± 3.06 ^Aab^	60.31 ± 3.32 ^Aab^	59.67 ± 1.79 ^Aab^	58.89 ± 1.62 ^Ab^
	DFC2	62.38 ± 3.18 ^Aa^	62.12 ± 2.24 ^Aa^	60.52 ± 3.56 ^Aab^	59.82 ± 2.80 ^Ab^	59.49 ± 2.93 ^Ab^	59.04 ± 3.96 ^Ab^
	SOC2	61.78 ± 4.73 ^Aa^	59.91 ± 3.33 ^ABab^	59.71 ± 1.64 ^Aab^	58.53 ± 4.10 ^ABb^	57.66 ± 3.92 ^ABb^	57.51 ± 5.34 ^Ab^
a*	NC	3.44 ± 0.63 ^a^	3.14 ± 0.55 ^ab^	2.93 ± 0.97 ^abc^	2.50 ± 0.76 ^bc^	2.17 ± 0.50 ^cd^	1.37 ± 0.50 ^Bd^
	DFC0	3.53 ± 0.50 ^a^	3.07 ± 0.33 ^ab^	3.01 ± 0.7 ^ab^	2.77 ± 0.58 ^bc^	2.13 ± 0.57 ^cd^	1.62 ± 0.49 ^Bd^
	DFC0.5	3.57 ± 0.54 ^a^	3.10 ± 0.55 ^ab^	2.79 ± 0.45 ^ab^	2.78 ± 0.60 ^ab^	2.37 ± 0.67 ^bc^	1.83 ± 0.66 ^Bc^
	DFC1	3.59 ± 0.38	3.10 ± 0.55	2.82 ± 0.65	2.95 ± 0.16	2.67 ± 0.64	2.49 ± 0.48 ^A^
	DFC2	3.55 ± 0.58 ^a^	3.35 ± 0.69 ^b^	2.95 ± 0.39 ^bc^	3.01 ± 0.50 ^bc^	2.82 ± 0.22 ^bc^	2.70 ± 0.52 ^Ac^
	SOC2	3.58 ± 0.68	3.19 ± 0.53	2.88 ± 0.56	2.83 ± 0.79	2.71 ± 0.66	2.54 ± 0.56 ^A^
b*	NC	11.07 ± 1.18 ^b^	11.57 ± 2.01 ^b^	11.94 ± 0.79 ^b^	12.28 ± 1.36 ^b^	12.71 ± 1.24 ^ab^	14.17 ± 1.22 ^Aa^
	DFC0	11.05 ± 2.04	11.88 ± 2.26	12.07 ± 0.53	12.12 ± 1.31	12.41 ± 1.99	12.97 ± 0.80 ^AB^
	DFC0.5	11.18 ± 2.02	11.95 ± 1.51	12.14 ± 1.63	12.70 ± 1.78	12.71 ± 1.99	13.08 ± 1.22 ^AB^
	DFC1	10.93 ± 2.06	10.54 ± 1.42	10.97 ± 1.25	11.35 ± 2.30	11.13 ± 2.36	11.68 ± 1.49 ^B^
	DFC2	10.81 ± 2.19	10.92 ± 1.12	11.01 ± 2.24	11.09 ± 0.96	11.11 ± 2.37	11.33 ± 0.68 ^B^
	SOC2	10.87 ± 1.44	10.70 ± 1.97	10.96 ± 1.98	11.15 ± 1.79	11.55 ± 2.36	11.84 ± 1.94 ^B^

^1)^ NC (non-coated), DFC0 (coated with duck fat, with no chitosan), DFC0.5 (coated with duck fat and 0.5% chitosan), DFC1 (coated with duck fat and 1% chitosan), DFC2 (coated with duck fat and 2% chitosan), SOC2 (coated with soybean oil and 2% chitosan). ^A–E^ Means values in the same column are significantly different (*p* < 0.05). ^a–e^ Means values in the same row are significantly different (*p* < 0.05). All values are presented as the mean ± SD of six replicates (*n* = 6).

**Table 2:** Microorganisms in coated chicken meat during storage at 4 ± 1 °C for up to 15 days.

Parameter (Log CFU/g)	Treatment ^1)^	Storage Period (Day)					
		0	3	5	7	10	15
**TVC**	NC	3.52 ± 0.06 ^e^	5.43 ± 0.11 ^BCd^	6.78 ± 0.04 ^Bc^	8.71 ± 0.02 ^Aa^	8.66 ± 0.05 ^Aa^	8.15 ± 0.01 ^Ab^
	DFC0	3.52 ± 0.06 ^e^	5.97 ± 0.02 ^Ad^	6.81 ± 0.05 ^Bc^	7.62 ± 0.02 ^Bb^	8.39 ± 0.22 ^Ba^	8.24 ± 0.05 ^Aa^
	DFC0.5	3.52 ± 0.06 ^f^	5.67 ± 0.02 ^ABe^	7.83 ± 0.02 ^Ad^	6.66 ± 0.26 ^Cc^	7.46 ± 0.09 ^Cb^	8.18 ± 0.04 ^Aa^
	DFC1	3.52 ± 0.06 ^e^	5.32 ± 0.28 ^Cd^	5.13 ± 0.02 ^Dd^	6.50 ± 0.01 ^Cc^	7.66 ± 0.03 ^Ca^	6.93 ± 0.04 ^Bb^
	DFC2	3.52 ± 0.06 ^c^	4.10 ± 0.17 ^Db^	4.02 ± 0.03 ^Eb^	4.24 ± 0.34 ^Eb^	6.32 ± 0.02 ^Ea^	6.15 ± 0.21 ^Ca^
	SOC2	3.52 ± 0.06 ^f^	5.24 ± 0.06 ^BCd^	5.48 ± 0.01 ^Cc^	4.85 ± 0.01 ^De^	7.14 ± 0.04 ^Da^	6.78 ± 0.01 ^Bb^
***E. coli***	NC	3.19 ± 0.06 ^e^	3.72 ± 0.34 ^ABd^	4.18 ± 0.07 ^Bc^	5.23 ± 0.12 ^Bb^	7.98 ± 0.04 ^Aa^	7.85 ± 0.09 ^Ba^
	DFC0	3.19 ± 0.06 ^f^	3.93 ± 0.04 ^Ae^	4.57 ± 0.10 ^Ad^	6.24 ± 0.02 ^Ac^	7.90 ± 0.02 ^Ab^	8.11 ± 0.06 ^Aa^
	DFC0.5	3.19 ± 0.06 ^e^	3.91 ± 0.19 ^Ad^	3.83 ± 0.09 ^Dd^	4.71 ± 0.10 ^Cc^	6.49 ± 0.02 ^Bb^	7.49 ± 0.02 ^Ca^
	DFC1	3.19 ± 0.06 ^e^	3.54 ± 0.09 ^ABCd^	3.92 ± 0.11 ^CDc^	4.07 ± 0.16 ^Dc^	6.58 ± 0.01 ^Ba^	6.32 ± 0.09 ^Eb^
	DFC2	3.19 ± 0.06 ^d^	3.20 ± 0.01 ^Ccd^	3.35 ± 0.01 ^Ecd^	3.39 ± 0.12 ^Ec^	4.36 ± 0.12 ^Db^	5.10 ± 0.04 ^Fa^
	SOC2	3.19 ± 0.06 ^e^	3.48 ± 0.01 ^BCd^	4.07 ± 0.01 ^BCc^	3.93 ± 0.21 ^Dc^	4.68 ± 0.06 ^Cb^	7.30 ± 0.06 ^Da^
**Coliform**	NC	3.10 ± 0.01 ^e^	3.72 ± 0.34 ^ABd^	4.15 ± 0.02 ^Bc^	5.19 ± 0.06 ^Bb^	7.95 ± 0.06 ^Aa^	7.83 ± 0.08 ^Ba^
	DFC0	3.10 ± 0.01 ^f^	4.16 ± 0.02 ^Ae^	4.71 ± 0.04 ^Ad^	6.13 ± 0.07 ^Ac^	7.95 ± 0.03 ^Ab^	8.08 ± 0.02 ^Aa^
	DFC0.5	3.10 ± 0.01 ^e^	3.80 ± 0.14 ^Ad^	3.86 ± 0.01 ^Cd^	4.69 ± 0.03 ^Cc^	6.41 ± 0.05 ^Bb^	7.42 ± 0.17 ^Ca^
	DFC1	3.10 ± 0.01 ^f^	3.81 ± 0.05 ^Ae^	3.93 ± 0.07 ^Cd^	4.18 ± 0.01 ^Dc^	6.57 ± 0.01 ^Ba^	6.36 ± 0.05 ^Db^
	DFC2	3.10 ± 0.01 ^c^	3.15 ± 0.21 ^Bc^	3.41 ± 0.01 ^Dc^	3.24 ± 0.34 ^Ec^	4.16 ± 0.17 ^Db^	5.14 ± 0.02 ^Ea^
	SOC2	3.10 ± 0.01 ^d^	3.76 ± 0.40 ^Ac^	4.13 ± 0.01 ^Bc^	3.78 ± 0.25 ^Dc^	4.76 ± 0.01 ^Cb^	7.41 ± 0.05 ^Ca^
**Yeast and molds**	NC	N.D.	1.00 ± 0.10 ^Cc^	3.77 ± 0.04 ^Ab^	4.17 ± 0.01 ^Aab^	4.75 ± 0.01 ^Aab^	5.47 ± 0.03 ^Aa^
	DFC0	N.D.	3.04 ± 0.01 ^Ad^	3.88 ± 0.02 ^Ac^	3.85 ± 0.01 ^Bc^	4.95 ± 0.07 ^Ab^	5.32 ± 0.03 ^Ba^
	DFC0.5	N.D.	2.15 ± 0.21 ^Bc^	3.63 ± 0.19 ^Ab^	3.66 ± 0.26 ^BCb^	3.69 ± 0.30 ^Cb^	4.65 ± 0.03 ^Ca^
	DFC1	N.D.	2.24 ± 0.34 ^Bc^	3.55 ± 0.03 ^Ab^	3.86 ± 0.06 ^Bb^	4.24 ± 0.05 ^Ba^	4.50 ± 0.03 ^Da^
	DFC2	N.D.	1.15 ± 0.36 ^Cb^	2.35 ± 0.49 ^Bab^	2.39 ± 0.12 ^Dab^	3.57 ± 0.05 ^Ca^	3.77 ± 0.03 ^Fa^
	SOC2	N.D.	2.60 ± 0.01 ^Bd^	3.78 ± 0.04 ^Ab^	3.41 ± 0.02 ^Cc^	3.82 ± 0.02 ^Cb^	4.01 ± 0.01 ^Ea^
***Listeria* spp.**	NC	N.D.	N.D.	N.D.	2.81 ± 0.47 ^Ab^	2.39 ± 0.12 ^Ab^	4.12 ± 0.23 ^Aa^
	DFC0	N.D.	N.D.	2.48 ± 0.01 ^Ab^	2.94 ± 0.34 ^Aab^	2.82 ± 0.31 ^Ab^	3.35 ± 0.16 ^Ba^
	DFC0.5	N.D.	N.D.	1.00 ± 0.10 ^Bbc^	3.38 ± 0.33 ^Aa^	2.15 ± 0.21 ^Aab^	3.35 ± 0.49 ^Ba^
	DFC1	N.D.	N.D.	N.D.	N.D.	1.24 ± 0.15 ^ABb^	3.93 ± 0.04 ^ABa^
	DFC2	N.D.	N.D.	N.D.	N.D.	N.D.	N.D.
	SOC2	N.D.	N.D.	N.D.	N.D.	N.D.	N.D.

^1)^ NC (non-coated), DFC0 (coated with duck fat, with no chitosan), DFC0.5 (coated with duck fat and 0.5% chitosan), DFC1 (coated with duck fat and 1% chitosan), DFC2 (coated with duck fat and 2% chitosan), SOC2 (coated with soybean oil and 2% chitosan). ^A–E^ Means values in the same column are significantly different (*p* < 0.05). ^a–f^ Means values in the same row are significantly different (*p* < 0.05). All values are presented as the mean ± SD of three replicates (*n* = 3).

## Data Availability

Data is contained within the article.
